# Thrombotic thrombocytopenic purpura with reversible splenial lesion syndrome: a case report

**DOI:** 10.1186/s12883-020-01696-2

**Published:** 2020-04-06

**Authors:** Song Hu, Xinyue Hou, Shuhao Liu, Chunxiao Fei, Lingyan Zhou, Ang Xing, Junqing Zhang, Chunming Yong, Xiaomeng Wang

**Affiliations:** The Affiliated Hospital of Qingdao University, Qingdao, Shandong China

**Keywords:** Thrombotic thrombocytopenic purpura, Reversible splenial lesion syndrome, Hemolytic uremic syndrome

## Abstract

**Background:**

Reversible splenial lesion syndrome (RESLES) is known to cause severe psychiatric symptoms but is also a very rare clinical disease in which the specific aetiology is unknown. According to current reports, there are major causes of the disease, including viral or bacterial infection, epilepsy, anti-epileptic drug withdrawal, high-altitude cerebral oedema, and metabolic disorders such as hypoglycaemia and hypernatraemia. In this article, we report a patient with thrombotic thrombocytopenic purpura (TTP) who presented with RESLES.

**Case presentation:**

A 34-year-old female patient who presented with fever and progression of disorder of consciousness was eventually diagnosed with RESLES based on brain imaging. Moreover, clinical features and peripheral smears demonstrating schistocytes and thrombocytopenia confirmed a diagnosis of TTP. RESLES can be improved by plasma exchange therapy.

**Conclusion:**

This rare case highlights the occurrence of RESLES as a presenting feature of the expanding list of unusual neurological manifestations of TTP.

## Background

Thrombotic thrombocytopenic purpura (TTP) is an invasive thrombotic microangiopathy classically defined by the pentad of fever, renal dysfunction, severe thrombocytopenia, haemolytic anaemia, terminal organ dysfunction and CNS involvement [1]. If not treated immediately, TTP can cause neurological symptoms such as consciousness disorders in 80% of patients and can have a 90% mortality rate. Multiple brain imaging abnormalities have been reported in patients with TTP [1]. Brain lesions were considered due to acute ischaemia if they exhibited a high-intensity signal on diffusion-weighted imaging and a corresponding low diffusion coefficient on the apparent diffusion coefficient map; cases were classified as posterior reversible encephalopathy syndrome (PRES) if they presented areas of vasogenic oedema with proven clinical or radiologic reversibility [2]. However, we report a patient with TTP who presented with reversible splenial lesion syndrome (RESLES).

## Case presentation

A 34-year-old previously healthy female presented with an 8-day history of fever and body aches, with a maximum body temperature increase at 38.5 °C. After administration of compound paracetamol, her symptoms did not improve. On the previous day, the symptoms were aggravated, with a reduced level of consciousness and a blunted response to conversation. Physical examination showed a body temperature of 39 °C. Petechiae and ecchymoses were scattered throughout her skin, and numerous blood scabs were observed in the mouth. She opened her eyes when saying her name and had slurred speech; the muscle strength of both lower limbs was level 3; muscle tension was moderate; abdominal wall reflex was present; bilateral knee and tendon reflex hyperactivity were present; lethargy was present; Gordon was positive; other pathological signs were not elicited; and meningeal irritation and bilateral Babinski’s signs were negative (GCS E3, V3, M4).

**Fig. 1:**
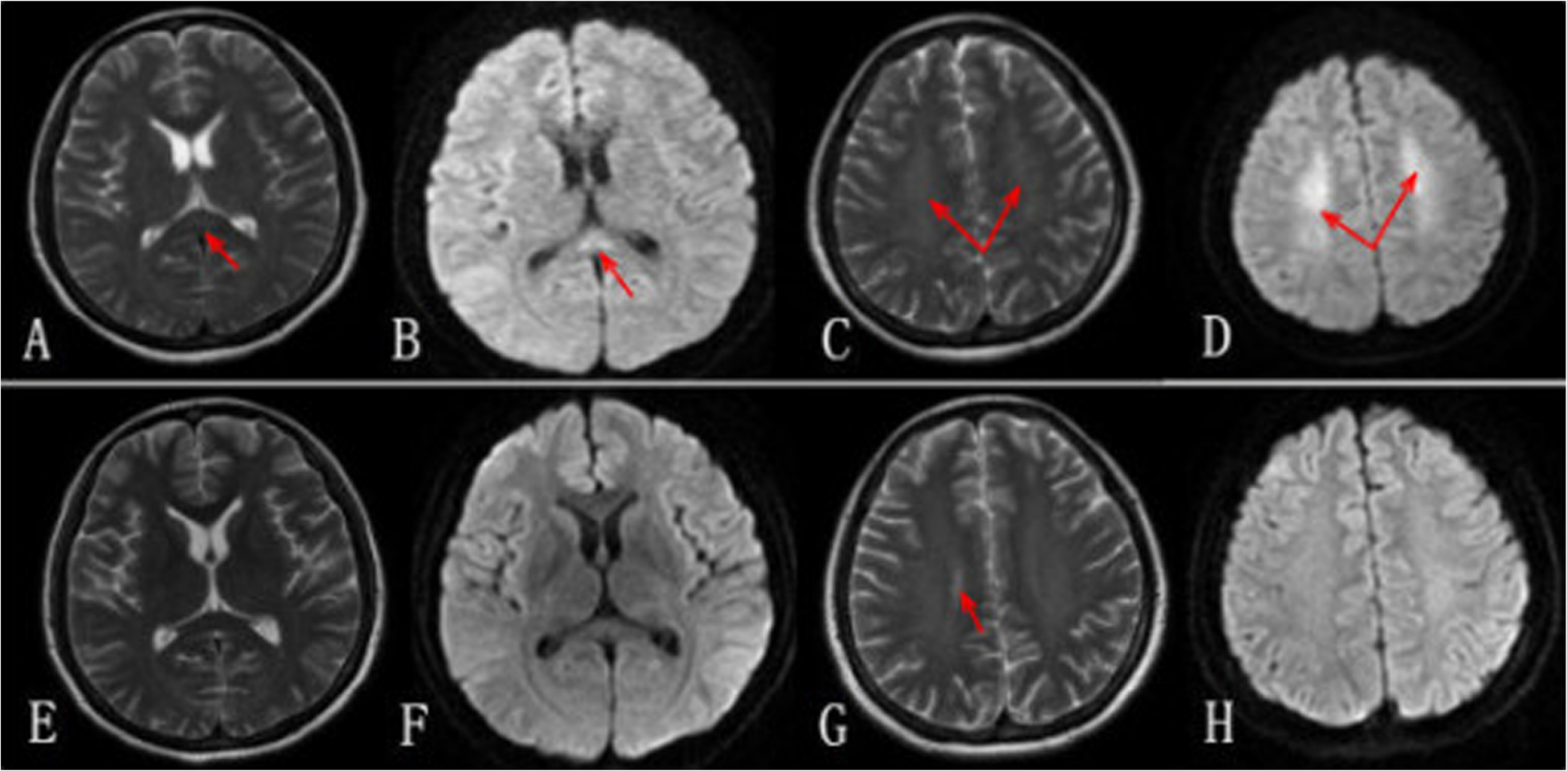
MRI on the day of admission (**a**, **b**, **c**, **d**) showed a focal high-signal lesion (arrow). After effective treatments (**e**, **f**, **g**, **h**), the splenial lesion had almost disappeared in the SCC

Abnormal indicators of peripheral blood analysis are shown in Table 1. The patient presented with schistocytes on a peripheral blood smear. Other blood biochemical tests (such as EB virus DNA, brucella, haemorrhagic fever and rheumatism) were normal. She underwent bone marrow biopsy, which showed an increased number of megakaryocytes. Cranial MRI showed a focal high-signal lesion involving the centrum semiovale, corona radiata and splenium of the corpus callosum (SCC) on diffusion-weighted and T2-weighted images (Fig. 1a, b, c, d). It takes into account reversible splenial lesion syndrome (RESLES type II). The chest, abdomen and pelvic CT did not have any abnormal phenomena. Initial clinic diagnosis included TTP, rhabdomyolysis, and anti-phospholipid antibody syndrome. The patient was immediately placed in the intensive care unit and treated with plasmapheresis, continuous renal replacement therapy (CRRT), and supplemented with fluid supplementation, correction of hyponatraemia, haemostasis, promotion of platelet production, meropenem anti-infection, acyclovir antiviral treatment, fibrinogen supplementation (cryoprecipitate infusion), plasma infusion, liver protection, etc. The clinical symptoms soon remitted, and she was discharged on day 20 after onset. After 2 weeks, the patient had no neurologic sequelae, and a follow-up MRI revealed significant regression of the lesion in the SCC (Fig. 1e, f, g, h).

**Table 1:** Abnormal indicators of peripheral blood analysis

Peripheral blood analysis	Initials	Abnormal index	Normal range
White blood cell count (/L)	WBC	2.80 × 10^9^	3.5–9.5 × 10^9^
Platelet (/L)	PLT	25.00 × 10^9^	100–300 × 10^9^
C-reactive protein (mg/L)	CRP	72.86	0–5
Procalcitonin (ng/mL)	PCT	15.63	<0.05
Creatine kinase (U/L)	CK	> 1600.0	6–80
Creatine kinase isoenzyme (U/L)	CKMB	37	0–6.6
Aspartate aminotransferase (U/L)	AST	1095.16	15–40
Alanine aminotransferase (U/L)	ALT	185.9	9–50
Serum creatinine (μmol/L)	SCR	119.5	31–132
Blood urea nitrogen (mmol/L)	BUN	11.9	3.6–9.5
Blood sodium (mmol/L)	Na+	132	137–147
D-Dimer (ng/mL)	–	23,750	0–500
Fibrinogen (g/L)	–	0.67	2–4
Plasma prothrombin time (sec)	PT	> 150.00	70–200
Activated partial thromboplastin time (sec)	APTT	48.7	22–38
Anti-cardiolipin antibody (IgM)	–	weakly positive	–
Anti-cardiolipin antibody (IgG + M + A)	–	weakly positive	–

Written informed consent was obtained from the patient for her data to be used anonymously for teaching and research purposes.

## Discussion

TTP and haemolytic uremic syndrome (HUS) are multisystem diseases that can be classified as primary thrombotic microangiopathy. Thus far, a total of 3 patients have been reported with HUS presenting with RESLES [3–5]. To the best of our knowledge, TTP presenting with RESLES has not been previously reported. TTP is strongly associated with cerebral and cardiac involvement in regard to central nervous system symptoms, which are mostly severe and mild, with a high mortality rate [4, 6, 7]. RESLES was proposed by Garcia-Monco [8] in 2011 as a new clinic-radiological syndrome. This syndrome is associated with a wide spectrum of diseases or a variety of factors, including acute encephalitis/encephalopathy syndrome, antiepileptic toxicity or sudden withdrawal, plateau cerebral oedema, metabolic diseases such as hypoglycaemia and hyponatraemia. However, the underlying pathophysiological mechanism remains unknown. Numerous studies [4, 9] have shown that RESLES is related to excitatory neurotoxicity, energy metabolic disorders, ion pump failure, and cell inside and outside water electrolyte imbalance interactions with cytotoxic brain oedema. The corpus callosum is susceptible to the above injury mechanism. Therefore, it can cause similar reversible corpus callosum focal lesions about one or more pathological mechanisms. The diagnosis mainly depends on the evolution of brain MRI. The acute stage lesions are usually elliptic, with clear boundaries and no obvious oedema or space-occupying lesions. The clinical manifestations of RESLES are diverse and unspecific.

This disease has been reported abroad and is not significantly different in the proportion of male and female patients. Takanashi et al. [10] summarized the common neurological symptoms, including delirium (54%), disturbance of consciousness (35%) and epilepsy (33%), by analysing the clinical manifestations of 54 patients with RESLES. In this report, the patient had symptoms associated with RESLES, such as consciousness disturbance and unresponsive response, and these clinical presentations were consistent with previous studies [10]. The patient had hyponatraemia after admission, and RESLES alone was clinically difficult to explain with hyponatraemia. It has been reported that encephalitis/encephalopathy patients with mild clinical symptoms of RESLES are often associated with hyponatraemia [8]; however, it is not clear why the cerebral oedema caused by low sodium is confined to the pressure of the corpus callosum. In this case, the patient was admitted with myalgia and fever, with high creatine kinase and inflammatory indicators and severe abnormal liver function. Unfortunately, the patient was transferred to the ICU and did not undergo EMG examination to further clarify the diagnosis due to serious illness. Acute liver dysfunction, high creatine kinase and malignant fever are associated with rhabdomyolysis, and this mechanism has been reported [11]. The increase in inflammatory factors and inflammatory cells caused cytotoxic oedema, suggesting that RESLES may be related to the activation of the immune system and the increase in some inflammatory response factors. TTP and HUS have been reported as potential trigger factors for PRES [2], and Burrus [1] pointed out that PRES is the most common imaging finding of moderate to severe TTP. In addition, PRES and RESLES are both unknown pathogenesis and imaging syndromes of reversible brain lesions. Accordingly, we speculate that RESLES is associated with TTP. Notably, this patient was weakly positive for anti-cardiolipin antibody and had higher D-dimer levels. After the above symptomatic treatment (consultation in each department, which included experts in rheumatism and immunity), the symptoms were obviously relieved. Previous reports have shown that TTP presents with anti-phospholipid antibody syndrome [12, 13], and Pan Yan et al. [9] reported that anti-phospholipid antibody syndrome may be the reason for RESLES. Hence, whether RESLES is two associated nodes to be further proved about the author’s point of view.

In summary, the author speculates that TTP may be a potential trigger factor for RESLES. This prompted us to propose a novel pathogenesis for RESLES, and we should correctly handle the relationships between and among TTP, RESLES, and anti-phospholipid antibody syndrome. However, further large-scale studies are necessary to confirm this theory.

## Data Availability

Not applicable.

## Abbreviations

TTP: Thrombotic thrombocytopenic purpura
HUS: Hemolytic Uremic Syndrome
RESLES: Reversible splenial lesion syndrome
PRES: Posterior Reversible Encephalopathy Syndrome
CT: Computed tomography
MRI: Magnetic resonance imaging
EB: Epstein-Barr
DNA: Defense Nuclear Agency
CRRT: Continuous renal replacement therapy
EMG: Electromyography
ICU: Intensive Care Unit
GCS: Grasse coma score
